# Differential Expression of MicroRNAs in the Mammary Gland of Lactating and Dry Murciano-Granadina Goats

**DOI:** 10.3390/ani16152357

**Published:** 2026-08-02

**Authors:** Mingjing Wang, Taina Figueiredo Cardoso, Antonia Noce, Endika Varela-Martínez, María Gracia Luigi-Sierra, Amparo Martínez, Juan Vicente Delgado, Jordi Jordana, Ahmed A. K. Salama, Xavier Such, Marcel Amills

**Affiliations:** 1 Centre de Recerca Agrigenòmica (CRAG), CSIC-IRTA-UAB-UB, Universitat Autònoma de Barcelona, 08193 Bellaterra, Spain; mingjing.wang@cragenomica.es (M.W.); noce.antonia.an@gmail.com (A.N.); endikavarela@hotmail.com (E.V.-M.); mgls@sund.ku.dk (M.G.L.-S.); 2 Departament de Ciència Animal i dels Aliments, Facultat de Veterinària, Universitat Autònoma de Barcelona (UAB), 08193 Bellaterra, Spain; jordi.jordana@uab.cat; 3 EMBRAPA Southeast Livestock, São Carlos, São Paulo 13560-970, Brazil; tainafcardoso@gmail.com; 4 Departamento de Genética, Universidad de Córdoba, 14071 Córdoba, Spain; ib2mamaa@uco.es (A.M.); juanviagr218@gmail.com (J.V.D.); 5 Group of Research in Ruminants (G2R), Department of Animal and Food Science, Universitat Autònoma de Barcelona (UAB), 08193 Bellaterra, Spain; ahmed.salama@uab.cat (A.A.K.S.); xavier.such@uab.cat (X.S.)

**Keywords:** post-transcriptional control, mammary involution, milk synthesis and secretion, metabolism

## Abstract

In goats, the influence of microRNAs on the post-transcriptional regulation of genes involved in mammary development and remodeling and milk secretion has not been fully elucidated yet. In this study, we have obtained mammary biopsies from five lactating and four dry Murciano-Granadina goats and, subsequently, small RNA has been purified and sequenced, obtaining, on average, 11 million reads per sample with an 85% alignment rate. Bioinformatic analysis of the sequencing data revealed the expression of 342 unique microRNAs in the goat mammary gland, among which 32 were novel miRNAs. Moreover, we have determined that as many as 93 mature microRNAs are differentially expressed (fold-change ≥ 2.7, q-value = 0.05) in lactating vs. dry goats. Of these, 49 and 44 were upregulated and downregulated in lactating goats, respectively, including 13 novel miRNAs. For each one of these differentially expressed miRNAs, we have made a search of scientific literature to find out whether their functional role in lactation has been elucidated, and, if so, we have contrasted the expected (based on function) vs. the observed (inferred from the differential expression analysis) change in gene expression to determine their degree of concordance. About 82% of the miRNAs with functional information that were differentially expressed in lactating vs. dry goats showed concordance between the observed and expected directions of the change in gene expression. By integrating differential expression analysis and functional data generated by others, we have identified a set of 17 miRNAs with converging evidence of being involved in the physiological transformations associated with lactation and mammary involution.

## Simple Summary

In goats, lactation and mammary involution during the dry period involve dramatic changes in the expression of protein-coding genes in the mammary gland. Part of these changes are driven by microRNAs, which have a repressive effect on the expression of protein-coding genes. In this study, we identify multiple microRNAs that are differentially expressed in the mammary gland of lactating vs. dry goats. We also show that, according to the existing literature, several of these microRNAs are involved in metabolic and regulatory functions crucial for milk synthesis and mammary cell proliferation and differentiation.

## 1. Introduction

In the mammary gland, lactation induces complex physiological changes driven by multiple environmental, epigenetic and genetic factors modifying gene expression. Such changes are partly driven by small non-coding RNA transcripts (~22 nucleotides in length), denominated microRNAs (miRNAs), that are expressed in the mammary gland [1]. These molecules are produced from longer primary miRNA precursors and exert their function at the post-transcriptional level by binding messenger RNAs (mRNAs) and repressing their expression through selective cleavage or translation repression [2].

Few studies have addressed the differential expression of mammary miRNAs in lactating vs. dry goats [3,4,5], providing evidence that lactation and mammary involution and remodeling involve changes in the expression of multiple miRNAs. Sequencing of the goat mammary transcriptome by Li and coworkers [3] revealed the expression of 346 conserved and 95 novel miRNAs, while in a subsequent study the number of miRNAs expressed in the goat mammary gland increased to 4038, 2988 of which were expressed in the three production cycle stages under study (late lactation, dry period and late gestation) [4]. In addition, a total of 754 goat mammary miRNAs were differentially expressed across the three physiological stages analyzed [4]. Among these, 185 miRNAs were upregulated and 247 were downregulated in the late-lactation versus dry-period comparison [4], highlighting that the transition from lactation to mammary involution involves major shifts in miRNA expression. In a more recent study, 1120 miRNAs were identified as expressed in the goat mammary gland, including 408 known and 712 novel miRNAs [5]. Amongst them, 62 and 190 miRNAs were differentially expressed in the early lactation vs. dry period and late lactation vs. dry period contrasts, respectively, thus highlighting that the expression profiles of mammary miRNAs across different production stages are remarkably dynamic [5]. In parallel, the biological roles of specific mammary miRNAs have been elucidated by performing functional studies [1]. For instance, miR-200a has been reported to inhibit the expression of genes involved in fat droplet formation [6], miR-24 is known to modulate triacylglycerol synthesis by interacting with fatty acid synthase [7], and miR-380-3p promotes β-casein expression [8].

In goats, the results of differential expression miRNA studies (comparing lactating vs. dry goats) and those obtained through the functional analysis of specific miRNAs have not been fully integrated yet. Such integration would be essential to further test the hypothesis that miRNAs have a key role in goat lactation and mammary involution and remodeling. The aim of the current work was to identify miRNAs expressed in the goat mammary gland that might be potentially involved in the regulation of genes with key biological roles in lactation and mammary involution. To do so, we have sequenced the small RNA fraction extracted from the mammary gland of 5 lactating and 4 dry Murciano-Granadina goats, and we have carried out a differential miRNA expression analysis. Moreover, we have integrated such analysis with published data about the functional roles of caprine mammary miRNAs by analyzing if such roles match the direction of the change in miRNA expression detected in the differential expression analysis.

## 2. Materials and Methods

### 2.1. Approval of Animal Procedures

The experimental protocols for performing mammary biopsies (procedure CEEAH 3859) and for euthanizing goats (procedure CEEAH: 4790) were approved by the Ethics Committee on Animal Experimentation at the Universitat Autònoma de Barcelona.

### 2.2. Animal Material and Sampling

Five Murciano-Granadina goats (average age 5.1 yr) were managed under identical husbandry conditions at the Experimental Fields and Farm Service of the Universitat Autònoma de Barcelona. Animals were housed at a stocking density of 1.5 m^2^ per goat and maintained under uniform environmental and management conditions. During winter and spring, goats grazed for 6 h daily on cultivated Italian ryegrass (*Lolium multiflorum* L.; Dry Matter 16.9%, Crude Protein 30.7%, Neutral Detergent Fiber 50.0%, Acid Detergent Fiber 24.9%, on a Dry Matter basis), whereas in autumn they grazed on Mediterranean natural pastures and shrubland. Indoors, goats were offered a total mixed ration ad libitum (forage:concentrate ratio 60:40% on a Dry Matter basis) consisting of alfalfa hay and a farm-mixed concentrate [ingredients (as fed): barley grain 37.0%, corn grain 15.0%, soybean hulls 15.0%, gluten feed 10.0%, soybean meal 5.0%, rapeseed 00 meal 5.0%, oat grain 4.5%, sunflower meal 2.0%, soybean oil 2.0%, cane molasses 2.0%, dicalcium phosphate 1.0%, calcium carbonate 0.5%, Vitafac Ovino-0.3 premix (DSM Nutritional Products, Barcelona, Spain) 0.5%, and salt 0.5%]. Water and commercial micromineral blocks (Multi-Block, Agrària Comarcal del Vallès, Llerona, Spain) were available ad libitum.

Goats were sampled during their lactation period (118 days after parturition). The mammary tissue from these five goats was retrieved with SPEEDYBELL 14G 150 mm semi-automatic biopsy needles (EVEREST Veterinary Technology, Barcelona, Spain) after administering 1 mL of lidocaine (20 mg/mL) to the udder area to be biopsied. We also extracted mammary biopsies from 4 goats that were slaughtered approximately 372 days after parturition, coinciding with day 98 of the dry-off period, due to reasons unrelated to our project. To minimize pain, goats were administered pentobarbital (150 mg/kg) in the jugular vein by a veterinarian. A lethal intravenous dose of sodium pentobarbital causes a goat to lose consciousness within seconds and results in clinical death within minutes. Subsequently, mammary samples were obtained by dissecting the udders of the slaughtered goats with a scalpel, a pair of scissors, and dissection forceps. Immediately after biopsy, all mammary tissue samples were submerged in RNAlater reagent (Thermo Fisher Scientific, Barcelona, Spain) and stored at −80 °C until use.

### 2.3. Purification and Sequencing of Small RNA

We extracted small RNA from the mammary samples by using the mirVana miRNA Isolation Kit (ThermoFisher, Sant Cugat del Vallès, Spain, https://www.thermofisher.com/order/catalog/product/es/en/AM1560 accessed on 22 November 2021) following the instructions of the manufacturer. An Agilent 2100 Bioanalyzer was used to measure the RNA Integrity Number (RIN), a value that indicates the quality of an RNA sample on a scale of 1 (degraded) to 10 (intact). The sequencing of the small RNA fraction was carried out in the Centre Nacional d’Anàlisi Genòmica (CNAG, https://www.cnag.eu/). Lactating and dry samples were processed simultaneously. Libraries were prepared using the NEBNext Small RNA Library Prep Set kit (Illumina, San Diego, CA, USA) according to the manufacturer’s protocol. Briefly, RNA was subjected to adapter 3’and 5’ ligation and first-strand cDNA synthesis. Afterwards, DNA fragments that had adapter molecules on both ends were selectively enriched by PCR. For library amplification, we used NEBNext Multiplex Oligos for Illumina, i.e., Index Primers Set 1 (ref. E7335), Index Primers Set 2 (ref. E7500), Index Primers Set 3 (ref. E7710) and Index Primers Set 4 (ref. E7730). With this approach, adapters with different indexes are used to sequence several samples in a single lane. All purification steps were performed with the AgenCourt AMPure XP beads (Beckman Coulter, L’Hospitalet de Llobregat, Spain). Libraries were evaluated with an Agilent Bioanalyzer (Agilent Technologies, Barcelona, Spain) to check the size distribution and concentration of library inserts. A pool was built to perform size selection using 6% Novex TBE PAGE gels (Thermo Fisher Scientific, Sant Cugat del Vallès, Spain), and subsequently the final pool was quantified by real-time quantitative PCR using the KAPA Library Quantification kit (Kapa Biosystems, Wilmington, MA, USA) prior to clonal cluster generation with an Illumina cBot instrument (https://emea.support.illumina.com/sequencing/sequencing_instruments/cbot.html accessed on 26 November 2025). Finally, all nine libraries were individually sequenced by generating single-end 1 × 50 bp reads with a HiSeq 2500 instrument (Illumina, San Diego, CA, USA).

### 2.4. Bioinformatic Analysis of the Sequencing Data

After sequencing, data were quality-checked using the software FastQC version 0.11.9 (https://www.bioinformatics.babraham.ac.uk/projects/fastqc/ accessed on 26 November 2025). The Trim Galore v 0.6.10 program was used to filter out low-quality reads (mean Q-score < 30) as well as reads with a size below 15 nucleotides. With the Trim Galore software v0.6.10, we allowed an error rate of 10% for adapter sequence matching and removed reads that had more than 2 ambiguous bases. MicroRNA detection and expression profiling were carried out with miRDeep2 (v 2.0.1.3). This package includes three modules, i.e., the miRDeep2 module which identifies known and novel miRNAs by analyzing next generation sequencing data; the Mapper module that, in our case, was used to align clean reads to the goat ARS1.2 (GCF_001704415.2) reference genome [9]; and the Quantifier module which maps the deep sequencing reads to predefined miRNA precursors and determines the expression of the corresponding miRNAs [10]. Reads shorter than 18 nucleotides or containing non-standard bases were filtered out to ensure data quality. The mature and precursor reference sequences included modified versions of the mature.fa and hairpin.fa files, in which uracil residues were converted to thymines to ensure compatibility with the pipeline. In the miRDeep2 database, cow (Bos taurus, -t bta) was used as a proxy for miRNA expression quantification. We decided to use this proxy because in miRbase, which is used by miRDeep2 to download known mature and precursor miRNA sequences, there are 1064 entries for *Bos taurus*, while the annotation for *Capra hircus* is rather poor, with just 267 entries. Novel miRNAs predicted with miRDeep2.pl were further filtered according to strict criteria, i.e., miRDeep2 score ≥ 6, estimated probability ≥ 90%, Rfam alert not associated with ribosomal RNA (rRNA) or transfer RNA (tRNA), significant randfold *p*-value (marked as “yes”), and mature read count ≥ 20. Only the novel miRNA candidates that met all the above criteria were classified as high-confidence novel miRNAs. Only miRNAs with expression levels above 0 raw counts in at least four samples were retained for quantification. This low threshold of expression was chosen because, in general, the expression of miRNAs is quite weak [11].

We used DESeq2 v1.44.0 [12] to identify miRNAs that were differentially expressed in the mammary gland of lactating vs. dry goats. We only took into consideration miRNAs that were expressed (read count > 0) in at least four samples and that had an average expression across samples greater than 20 read counts. Since sample size was an important constraint, which might limit our ability to reliably detect changes in miRNA expression and to generalize our findings, we followed the indications of Schurch et al. [13], who showed that with 3 replicates per group only 20–40% of the significantly differentially expressed genes identified with a full set of 42 clean replicates could be detected, but that such percentage increased to 85% for genes with 4-fold differences in mRNA expression. To infer which fold-change value can be reliably detected, in our conditions, with a statistical power of 80%, we used the RNASeqPower package [14] (https://bioconductor.org/packages/release/bioc/html/RNASeqPower.html accessed on 26 November 2025). As input data, we introduced (1) an average sample number of 4.5 per group; (2) an average coefficient of variation (CV) of 0.52; (3) a sequencing depth of 53,526 calculated as follows: 11 million reads per sample × 0.85 alignment rate × 50 nucleotides (nt) per read = 467,500,000 nt and miRNA total space = 397 miRNA × 22 nt = 8734 nt, so sequencing depth = 467,500,000 nt/8734 nt = 53,526; (4) an average statistical power of 80%; and (5) an α-value of 0.05. Under these conditions, a statistical power of 80% could be achieved when detecting differential expression at an absolute fold change (FC) value of 2.7 or higher (absolute log_2_FC ≥ 1.43 or higher). Thus, we considered that there was a significant differential expression when log_2_FC ≥ 1.43 and the *q*-value, calculated with the false discovery rate approach [15], was lower than 0.05.

A structured literature search was performed to identify functional information associated with differentially expressed miRNAs. This process was complemented by the use of two large language models (ChatGPT-4o and Gemini 1.5 Pro) as exploratory tools to assist in the identification of candidate miRNAs previously linked to lactation. The input provided to these tools consisted of the list of differentially expressed miRNAs obtained in this study, and they were prompted to identify miRNAs with reported roles in lactation-related processes. All outputs generated by the AI tools were manually reviewed, and only those supported by peer-reviewed primary literature were retained. AI-derived suggestions were used exclusively as an aid for literature screening and did not influence data analysis or interpretation.

## 3. Results

The average RIN score of the RNA extracted from mammary samples retrieved from five lactating and four dry goats was 7.67 ± 0.67. Small RNA sequencing of these samples yielded an average of 11,151,376 raw single-end 1 × 50 bp reads per sample (Supplementary Table S1). On average, the mapping rate was 85% and 9,308,021 reads were successfully aligned to the NCBI goat ARS1.2 (GCF_001704415.2) reference genome [9] (Supplementary Table S1). Analysis of the sequencing data with the miRDeep2 (v 2.0.1.3) software package [10] made it possible to identify 397 mature miRNAs that were expressed in the goat mammary gland of at least four goats after filtering out lowly expressed miRNAs (Supplementary Table S2). After accounting for precursor redundancy, these corresponded to 342 unique mature miRNAs, including 32 novel miRNAs (Supplementary Table S2).

**Figure 1:**
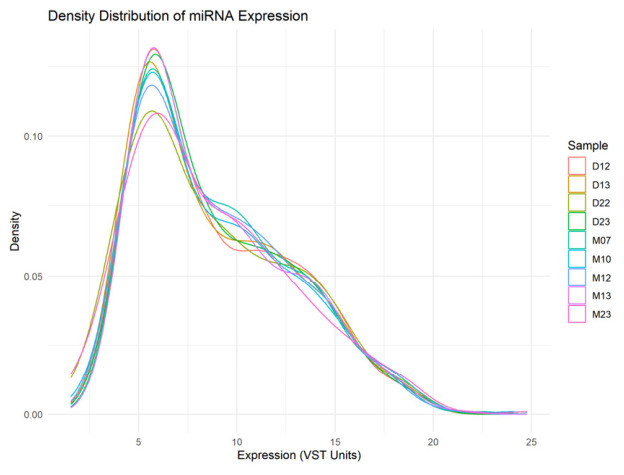
Distribution of the expression levels of 342 miRNAs in each mammary sample. Raw read counts have been transformed with the Variance Stabilizing Transformation (VST) function implemented in DESeq2. The density curves correspond to individual samples shown in the legend on the right; samples with the prefix D represent mammary tissue from dry goats, while samples with the prefix M represent mammary tissue from lactating goats. The distribution of transformed read counts is strongly skewed to the left, thus evidencing that most miRNAs have low levels of expression.

In Figure 1, we show the distribution of the average levels of expression of miRNAs by transforming read counts with the Variance Stabilizing Transformation function implemented in DESeq2 [12]. With this approach, the variance becomes (approximately) independent of the expression average while expression values can be normalized with respect to library size [16].

**Figure 2:**
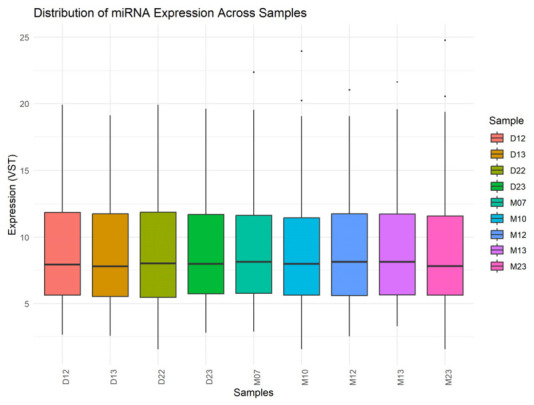
Boxplots indicating the median expression levels of miRNAs transformed with the VST function. D and M prefixes correspond to dry and lactating samples, respectively.

The expression levels of miRNAs in each goat mammary sample are depicted in Figure 2.

**Figure 3:**
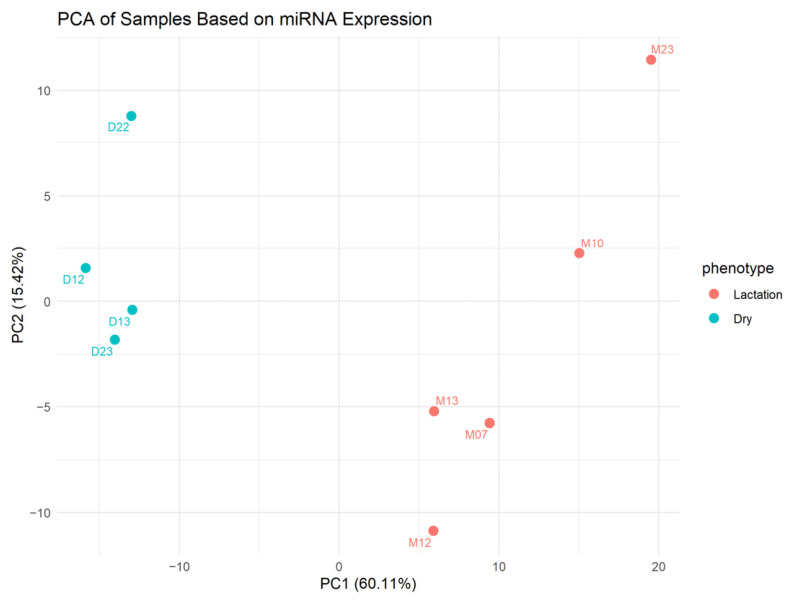
Principal component analysis of 9 mammary samples from dry and lactating goats based on their miRNA expression profiles. Dry and lactating samples are indicated with D and M prefixes.

Principal component analysis of miRNA expression profiles from the nine goat mammary tissue samples under study evidenced a clear separation between the lactating and dry groups (Figure 3).

**Figure 4:**
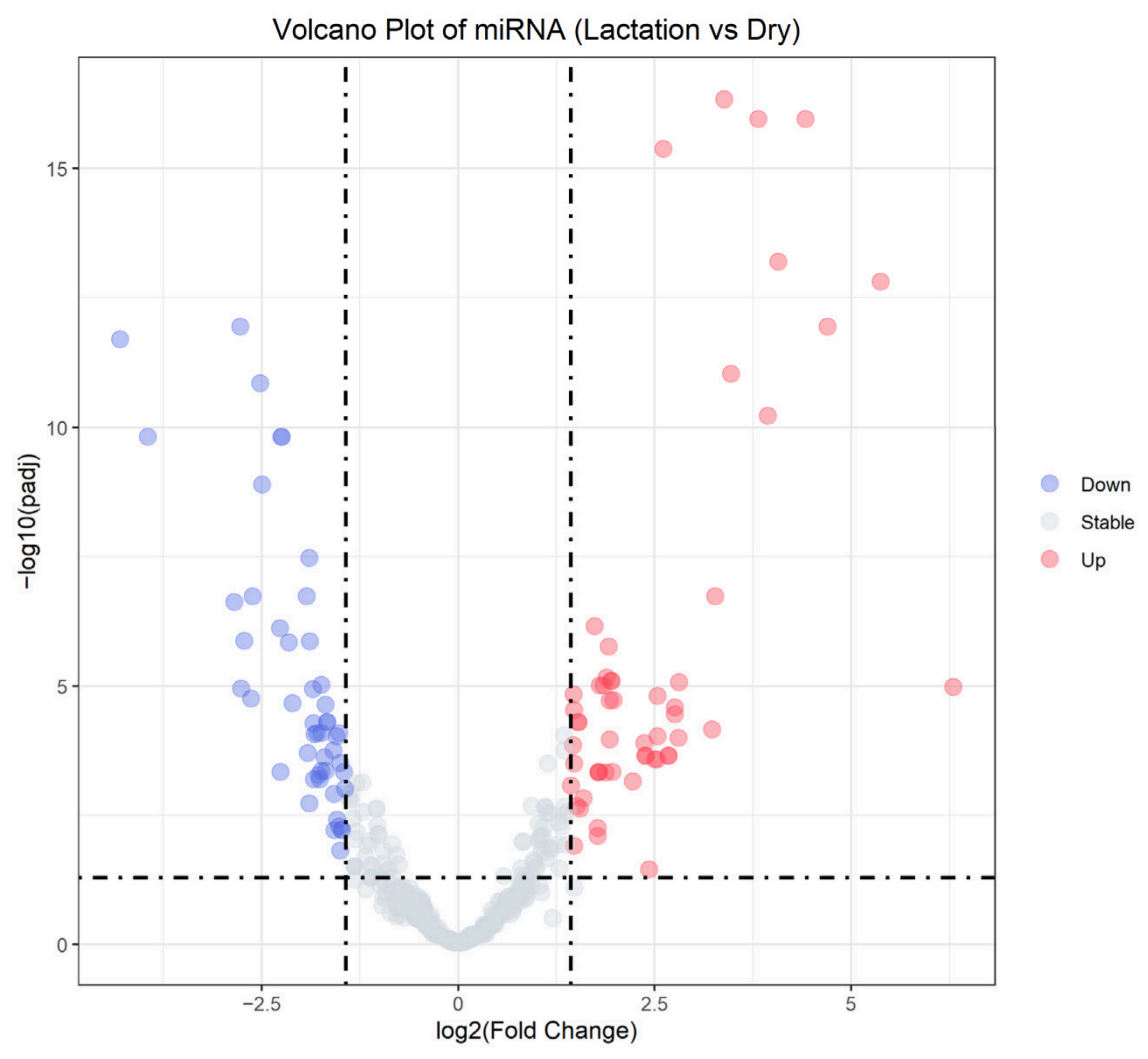
Volcano plot showing miRNAs that are differentially expressed in the mammary gland of lactating vs. dry goats. Red and blue dots represent miRNAs that are upregulated and downregulated in lactating goats, respectively. The vertical and horizontal dashed lines indicate the log_2_fold-change and *q*-value (padj) thresholds, respectively, at which a miRNA is considered to be differentially expressed.

To further explore such differences, we investigated the differential expression of miRNAs in these two physiological states. By doing so, we identified 104 miRNAs (13 novel miRNAs) that were differentially expressed, of which 55 and 49 were upregulated and downregulated in lactating goats, respectively (Figure 4, Supplementary Table S3).

When we took into account that identical miRNAs can originate from different precursors due to gene duplication events and other factors, this list of differentially expressed miRNAs was reduced to 93 miRNAs, of which 49 (11 novel miRNAs) were upregulated and 44 (2 novel miRNAs) were downregulated in lactating goats (Figure 4, Supplementary Table S3).

In order to infer the biological significance of the results obtained in the miRNA differential expression analysis, we have explored the functional roles of differentially expressed miRNAs by performing a comprehensive in-depth literature search for each differentially expressed miRNA. By doing so, we have identified 21 miRNAs that showed differential expression between dry and lactating goats and for which functional information is available (Table 1). Although it is possible that we might have missed some relevant miRNAs in our search, this list of 21 miRNAs was representative enough to evaluate whether the observed change in miRNA expression (inferred from the differential expression analysis) was concordant with the predicted change in gene expression based on functional information. Overall, about 82% of the miRNAs yielded consistent results (Table 1). We found a discrepancy between the observed and expected effects for bta-miR-23a and bta-miR-135a, while for bta-miR-142-5p and bta-miR-378 the degree of discrepancy was less clear due to conflicting results about the function of these two miRNAs in the literature. This high concordance between previously published functional evidence and our differential expression results indicates that our analysis captures a genuine biological signal and is therefore unlikely to be strongly affected by technical or experimental biases.

## 4. Discussion

In our study, we identified 342 unique mature miRNAs expressed in the goat mammary gland, including 32 novel miRNAs. In contrast, Xuan et al. [4] identified as many as 4038 miRNAs that were expressed in the mammary tissue of goats, but this number decreased to 1120 in a later study performed by Peng et al. [5]. In mice, Confuorti et al. [39] reported the expression of 419 and 460 miRNAs in the mammary gland of lactating and involuting females, a number closely aligned with ours, and most of them were shared by both groups of individuals. Moreover, the analysis of 28,866 human small RNA sequencing data sets combined with the performance of validation experiments made it possible to establish that in humans there are approximately 2300 mature microRNAs [40], a figure that is much lower than the number of miRNAs that Xuan et al. [4] reported as expressed in the goat mammary gland.

Examination of Figure 1 shows that the distribution of transformed read counts is strongly skewed to the left, evidencing that most miRNAs have low levels of expression. Such findings are consistent with the distribution of miRNA expression data reported by other authors [41], which is also skewed to the left. For instance, Gong et al. [11] analyzed 410 human RNA-Seq datasets representing a broad diversity of tissues and found that on average ~70% of known miRNAs were expressed at low levels or not expressed (RPM < 1) in a sample, and only ~9% of known miRNAs were highly expressed (RPM > 100). Overall, the distribution of miRNA expression (Figure 2) is relatively consistent across samples, suggesting comparable sequencing depth and stable miRNA expression profiles. The principal component analysis (Figure 3) of the nine goat mammary samples based on their profile of miRNA expression highlighted the existence of substantial differences between the miRNA expression profiles of lactating and dry goats.

We detected 93 miRNAs that showed differential expression between lactating and dry goats, of which 49 were upregulated and 44 were downregulated in lactating goats (Figure 4). Several aspects of our experimental design warrant consideration to properly contextualize such findings. First, a remarkable limitation of our study is that samples from lactating goats were retrieved by biopsy while those from dry goats were extracted by euthanasia and tissue dissection. Differences in the method of collection were dictated by management constraints, and we acknowledge that this could be a source of experimental noise. However, both procedures targeted the same anatomical region and yielded comparable tissue types. Moreover, tissue retrieval from dry goats was carried out a few minutes after death, so ischemia is not expected to have a strong effect on expression patterns. Indeed, prolonged cold ischemic delay of human breast cancer tissue samples up to 3 h produced marginal global mRNA expression changes [42], and similar results have been obtained by Ferreira et al. [43] for GTEx tissues, by showing that most alterations in mRNA expression take place between 7 and 14 h post-mortem. Even more, there is compelling evidence that miRNAs are particularly resistant to post-mortem degradation, due to their small size and shielding by Argonaute proteins, so their post-mortem expression remains stable for several hours and, even, for days [44,45,46]. Indeed, the exceptional stability of miRNAs has made them ideal tools in forensic science to estimate time since death over hours to days [45]. A second important consideration is that we were unable to validate the differential expression results by real-time quantitative RT-PCR because the amount of available biological material was insufficient, a circumstance that makes it difficult to experimentally estimate the false positive rate. However, we compared the direction of the observed change in miRNA expression between lactating and dry goats with the expected change inferred from functional evidence. Because concordance is not guaranteed and may be low, this represents a stringent test; thus, the high level of agreement observed supports the biological relevance of our findings.

A third key aspect to consider is that we did not carry out an in silico prediction of the potential mRNA targets of differentially expressed miRNAs, because there is compelling evidence that miRNA target prediction programs suffer from high false positive rates [47]. While computational miRNA target prediction tools typically rely on identifying phylogenetically conserved seed matches within gene 3’UTRs, in many cases the 3’UTR binding sites are more strongly conserved than the miRNA seeds themselves, raising concerns about the true biological significance of these strongly conserved sites [47]. Based on this observation, Pinzón and coworkers estimated that the false positive rate for tools such as microT and miRanda might approach 50–70% [47]. In an independent study, Fridrich and coworkers compared the number of predicted miRNA targets to the number of targets that were experimentally validated in a HEK293 cell line and in the sea anemone *Exaiptasia pallida*, and found that ~65% and ~85% of the predicted targets in HEK293 and *Exaiptasia*, respectively, were artifacts [48]. Such a flaw might substantially bias the results of Gene Ontology enrichment analyses and might lead to wrong conclusions about the functional significance of differentially expressed miRNAs [48].

### 4.1. Differential Expression of microRNAs with Effects on the Expression of Transcriptional Regulators and Caseins

In lactating goats, we have detected the downregulation of bta-miR-221 (Table 1 and Table S3). This miRNA represses the expression of the signal transducer and activator of transcription 5A (*STAT5A*) and insulin receptor substrate 1 (*IRS1*) mRNAs [34], so its downregulation is consistent with the key role of the STAT5A transcription factor to promote mammary lobuloalveolar outgrowth and lactogenesis [49], while the loss of IRS1 is associated with a decreased lactation capacity and reduced pup weight [50].

Prolactin is a hormone produced by the anterior pituitary with mammogenic and lactogenic effects, being essential for the maintenance of lactation [51] and the synthesis of α_S1_- and α_S2_-caseins [52,53]. Interestingly, we have detected that bta-miR-138, which inhibits the translation of the prolactin receptor (PRLR) by regulating STAT5 and MAPK [24], is downregulated in lactating goats. Obviously, the downregulation of bta-miR-138 (Table 1 and Table S3) is consistent with the increased expression of PRLR during lactation.

The downregulation of bta-miR-15a in lactating goats (Table 1 and Table S3) agreed well with its inhibitory impact on the expression of the growth hormone receptor and the casein genes [17]. Transfection experiments in bovine mammary epithelial cell-derived MAC-T cells have demonstrated that growth hormone upregulates the expression of the α_S1_-casein, α_S2_-casein, β-casein, and α-lactalbumin genes [54]. Moreover, growth hormone deficiency causes a reduction in milk yield due to the loss of secretory cells [55]. Noteworthy, the upregulation of bta-miR-30d in lactating goats was consistent with a report indicating that this miRNA is associated with an increased content of milk proteins and caseins [18].

For one miRNA (Table 1 and Table S3), we observed a discrepancy between the results of the differential expression analysis and functional data. In lactating goats, bta-miR-135a was upregulated despite its repressive effect on PRLR expression [23]. The existence of discrepancies between the observed and the expected effects of miRNAs might be due to the occurrence of potential errors in either the differential expression analysis or the functional characterization of miRNAs, but it might also be explained by other causal factors. Indeed, miRNAs have the capacity to regulate the expression of hundreds of genes involved in distinct and unrelated biological processes [56]. For instance, miR-135a is involved not only in the regulation of PRLR but also in lipid metabolism [57] and immunity [58]. Besides that, the expression of a single mRNA can be directly or indirectly regulated by multiple miRNAs [59], and in some instances the expression of an mRNA could be determined by the equilibrium between repressive and enhancing miRNA-mediated signals as a cooperative mechanism to fine-tune it. Finally, there is evidence of a weak correlation between miRNA activity and expression [60] as well as of a high variability in the strength of correlations between miRNAs and their target genes [61]. It should also be noted that the repression exerted by miRNAs on gene expression is usually mild (i.e., less than twofold), so, in general, miRNAs are assumed to fine-tune rather than abrogate gene expression [47].

### 4.2. Differential Expression of microRNAs with Functions Related to Lipid Metabolism

We have also detected that several miRNAs known to repress the synthesis of milk fat are downregulated in lactating goats (Table 1 and Table S3), a finding that highlights the high concordance between the direction of the change in miRNA expression detected by us and that predicted by miRNA function. Examples of this are bta-miR-130b and bta-miR-454, which repress peroxisome proliferator-activated receptor γ expression [22,37]; bta-miR-150, which has an inhibitory effect on de novo lipogenesis [29]; bta-miR-183, which inhibits milk fat metabolism [31]; bta-miR-199a-3p, which targets the very low-density lipoprotein receptor [32]; and bta-miR-497, which impairs lipid metabolism [38].

Lactation also involved the upregulation of several miRNAs that promote lipid synthesis (Table 1 and Table S3). For instance, in goat mammary epithelial cells, bta-miR-148a, which is upregulated in lactating goats, induces triglyceride synthesis by suppressing the action of two genes, peroxisome proliferator-activated receptor α (*PPARA*) and peroxisome proliferator-activated receptor γ coactivator 1 α (*PPARGC1A*), which promote fatty acid β-oxidation [28].

In the case of bta-miR-142-5p [25], this miRNA has been reported to enhance triglyceride synthesis but, despite this, it was downregulated in lactating goats (Table 1 and Table S3). However, there is also evidence that this miRNA has a dual role in lipid metabolism because it has also been described as inhibiting SREBP1 [26], a result that would be consistent with its downregulation in lactating goats. Similarly, bta-miR-378 was upregulated in lactating goats, but to infer its expected effect is difficult because this miRNA has been reported to inhibit lipoprotein lipase (*LPL*) and *CD36* mRNA expression [35] and to stimulate lipogenesis [36]. Finally, bta-miR-23a was also downregulated in lactating goats, a finding inconsistent with its role in promoting the expression of *FASN*, *ADRP*, *CD36*, and *TIP47* mRNAs [6]. Reasons for these inconsistencies were mentioned in the previous section and will not be repeated here.

### 4.3. Differential Expression of microRNAs Controlling the Proliferation and Differentiation of Mammary Cells

There is substantial evidence that, in ruminants, mammary involution is characterized by the conservation of the lobuloalveolar structure accompanied by an intense cell turnover and cell proliferation [62]. Indeed, mammary cell proliferation is more intense during the dry period than during lactation [62]. In close agreement with this, we have observed that several miRNAs promoting cell proliferation, i.e., bta-miR-31 [19], bta-miR-106b [21] and bta-miR-182 [30] are downregulated in lactating goats (Table 1 and Table S3). Notably, bta-miR-31 enhances mammary epithelial proliferation by modulating multiple pathways, such as PRLR/STAT5, transforming growth factor β (TGFβ) and Wnt/β-catenin [19] signaling, while bta-miR-106b stimulates the proliferation and cell cycling of bovine mammary epithelial cells as well as the expression of protein synthesis-related pathways [21]. We have also found that bta-miR-34a, which induces apoptosis [20], is also downregulated in lactating goats, a finding consistent with the increased rate of apoptosis in the mammary gland during the dry-off period [62]. Moreover, bta-miR-146b was upregulated in lactating goats (Table 1 and Table S3), a finding consistent with its role in maintaining pregnancy-derived mammary luminal alveolar progenitors and repressing STAT3β and promoting β-casein mRNA expression [27].

### 4.4. Differential Expression of microRNAs Involved in Immunity

We have detected the downregulation in lactating goats of miRNAs with important roles in immunity (Supplementary Table S3), i.e., bta-miR-29b, which suppresses immunity against intracellular pathogens by targeting IFN-γ [63], as well as of bta-miR-146a, which regulates inflammatory cytokines of bovine mammary epithelial cells by interfering with the TLR4/TRAF6/NF-κB pathway [64,65]. In addition, we have found that bta-miR-122 and bta-miR-146b, which regulate the innate immune response against pathogens [66,67], and bta-miR-375, which modulates bovine mammary inflammation [68], are upregulated in lactating goats (Supplementary Table S3). Since there is no conclusive evidence to anticipate the degree to which immunity is reduced or augmented during the dry-off period, we have not attempted to link the observed changes in the expression of these miRNAs with those predicted based on functional data.

Interestingly, several of these miRNAs that show differential expression in lactating and dry goats also modify their expression in response to mastitis. Of special relevance is bta-miR-146a, which displays upregulated expression in mammary gland tissues with subclinical or clinical mastitis and, possibly, modulates proinflammatory pathways [69]. bta-miR-122 is likewise upregulated in *Streptococcus agalactiae*-induced bovine mastitis, suggesting a regulatory role in shaping the JAK–STAT signaling response during pathogen-driven inflammation [70]. Our observation that several of these proinflammatory miRNAs are downregulated in lactating goats is consistent with evidence obtained in cattle, showing that the early dry period involves systemic and mammary physiological inflammation, with increased percentages of CD20^+^ B lymphocytes, CD172a^+^ macrophages, and neutrophils in the mammary tissue [71].

## 5. Conclusions

In summary, we have demonstrated that 342 miRNAs are expressed in the caprine mammary gland and 93 of them show differential expression (absolute log_2_FC ≥ 1.43) when comparing lactating vs. dry goats. By performing an exhaustive literature-based functional integration, we obtained experimentally supported biological information for 21 of these miRNAs. Interestingly, we found a high concordance (≈82%) between the observed direction of differential expression and the expected regulatory effect inferred from their known functions. This integrative approach adds a strong layer of biological interpretation to the sequencing data and allows us to identify a core set of 17 miRNAs with converging evidence suggesting their involvement in the physiological changes associated with lactation and mammary involution. Further functional studies are required to determine the precise biological roles of these miRNAs in mammary physiology and to elucidate their regulatory mechanisms of action.

## Figures and Tables

**Table 1:** Concordance and discordance (shown in red) between the expected effects of miRNAs (based on functional data) and the observed changes in miRNA expression in lactating goats (based on differential expression analysis).

miRNA	Reference	Functional Effect	Expected	Observed	Observed log_2_FC
bta-miR-15a	[17]	Inhibits GHR and caseins	DOWN	DOWN	−1.78
bta-miR-23a	[6]	Promotes mRNA expression of *FASN*, *ADRP*, *CD36*, and *TIP47* mRNAs	**UP**	**DOWN**	−1.89
bta-miR-30d	[18]	Associated with increased milk protein and casein content	UP	UP	1.47
bta-miR-31	[19]	Promotes cell proliferation	DOWN	DOWN	−1.56
bta-miR-34a	[20]	Induces cell apoptosis	DOWN	DOWN	−2.57
bta-miR-106b	[21]	Promotes cell proliferation and cycling	DOWN	DOWN	−1.69
bta-miR-130b	[22]	Represses PPARG	DOWN	DOWN	−1.73
bta-miR-135a	[23]	inhibits PRLR	**DOWN**	**UP**	2.00
bta-miR-138	[24]	Inhibits PRLR	DOWN	DOWN	−4.25
bta-miR-142-5p	[25]	Promotes milk fat metabolism	**UP**	**DOWN**	−1.85
	[26]	Inhibits SREBP1	DOWN	DOWN	
bta-miR-146b	[27]	Promotes the maintenance of mammary luminal alveolar progenitors	UP	UP	2.64
bta-miR-148a	[28]	Promotes triacylglycerol synthesis	UP	UP	4.72
bta-miR-150	[29]	Represses de novo lipogenesis	DOWN	DOWN	−1.72
bta-miR-182	[30]	Enhances cell proliferation	DOWN	DOWN	−1.82
bta-miR-183	[31]	Inhibits milk fat metabolism	DOWN	DOWN	−1.89
bta-miR-199a-3p	[32]	Represses LPL, ACACA, FASN, SCD, CD36 and FABP3	DOWN	DOWN	−2.20
bta-miR-206	[33]	Suppresses triacylglycerol synthesis	DOWN	DOWN	−2.68
bta-miR-221	[34]	Represses STAT5A and IRS1	DOWN	DOWN	−1.80
bta-miR-378	[35]	Inhibits *LPL* and *CD36* mRNAs	**DOWN**	**UP**	1.93
	[36]	Increases lipogenesis	UP	UP	
bta-miR-454	[37]	Represses PPARG	DOWN	DOWN	−1.54
bta-miR-497	[38]	Impairs fat metabolism	DOWN	DOWN	−1.64

## Data Availability

Raw sequencing data can be accessed via PRJNA1250936 in the National Center for Biotechnology Information (NCBI). Supplementary Files can be accessed at: https://figshare.com/s/bc18686b7090018d92e4 (accessed on 06 June 2026).

## Supplementary Materials

The following supporting information can be downloaded at: https://www.mdpi.com/article/10.3390/ani16152357/s1, Table S1: Statistics of the small RNA sequencing experiment; Table S2: Expression levels of 397 miRNAs in dry and lactating goats (raw counts); Table S3: List of microRNAs that are differentially expressed between lactating and dry goats (lactating goats are used as reference to define upregulation and downregulation).

## Abbreviations

BRCA1	Breast Cancer 1, Early Onset
GHR	Growth Hormone Receptor
IGF1R	Insulin-like Growth Factor 1 Receptor
mRNAs	Messenger RNAs
PPARA	Peroxisome Proliferator-Activated Receptor α
PPARGC1A	Peroxisome Proliferator-Activated Receptor Gamma Coactivator 1α
PRLR	Prolactin Receptor
SREBF1	Sterol Regulatory Element Binding Transcription Factor 1
STAT5A	Signal Transducer and Activator of Transcription 5A
TGFβ	Transforming Growth Factor β
