# Supplementary material for: Differential Expression of MicroRNAs in the Mammary Gland of Lactating and Dry Murciano-Granadina Goats
Source: Animals (Basel). 2026 Aug 2;16(15):2357. doi: 10.3390/ani16152357 (PMC13463571; doi:10.3390/ani16152357)
Supplement: Supplementary file 1 [file animals-16-02357-s001.zip › Supplementary Table S1.pdf]

Supplementary Table S1. Statistics of the small RNA sequencing experiment

**Sample[1]**	**Raw reads**	**After trimming**	**Total mapping reads**	**Mapped reads**	**Mapped %**
D12	11,242,774	11,167,540	11,025,039	9,606,228	87.13
D13	12,201,323	12,109,984	11,957,393	10,403,101	87.00
D22	7,010,114	6,966,447	6,849,131	5,674,076	82.84
D23	10,761,903	10,661,290	10,491,104	9,270,060	88.36
M10	13,299,672	13,140,584	12,978,622	11,906,872	91.74
M12	10,830,796	10,713,746	10,517,272	9,253,178	87.98
M13	11,755,028	11,557,706	11,247,949	9,069,037	80.63
M23	12,009,657	11,909,116	11,773,087	10,807,921	91.80
M07	11,251,121	11,048,175	10,661,153	7,781,717	72.99
Average	11,151,376	11,030,510	10,833,417	9,308,021	85.61

[1]M and D samples correspond to lactating and dry samples, respectively. The total mapped reads were obtained by discarding sequences containing non-canonical bases and removing short reads during data processing.
